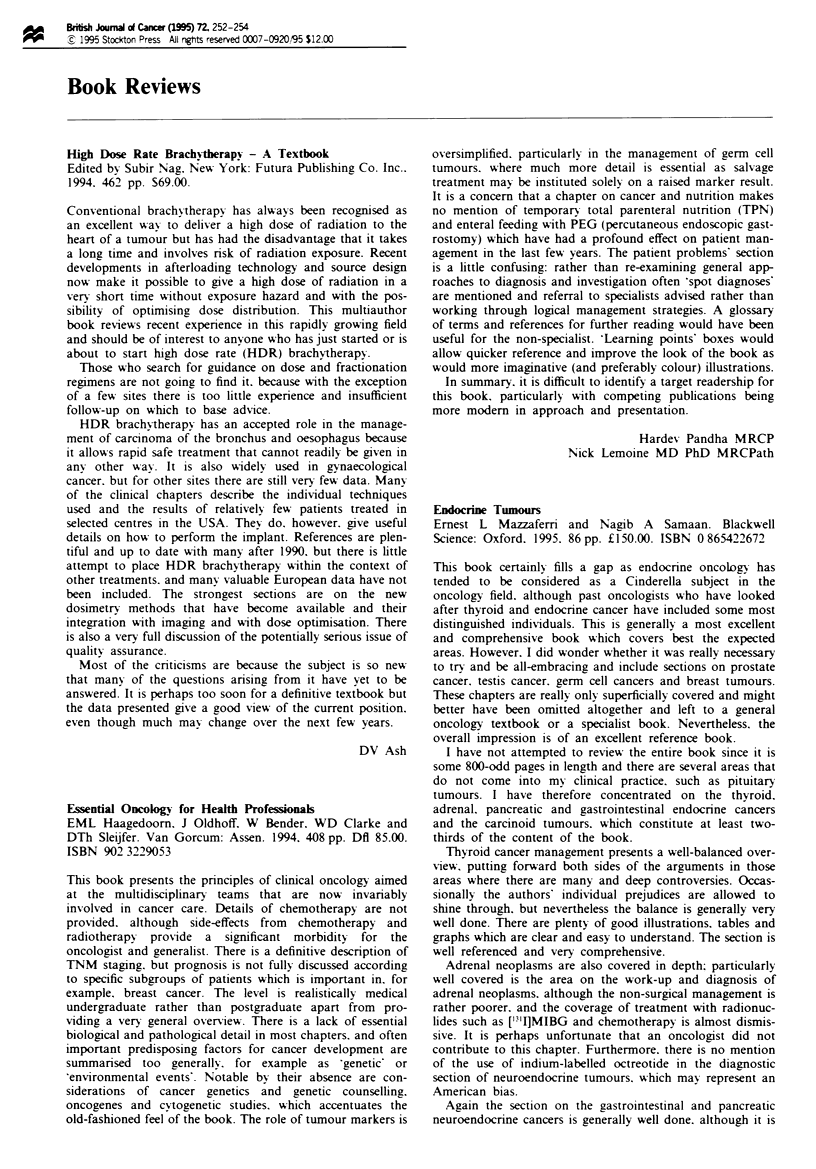# High Dose Rate Brachytherapy - A Textbook

**Published:** 1995-07

**Authors:** DV Ash


British Journal of Cancer (1995) 72, 252-254

© 1995 Stockton Press All rights reserved 0007-0920/95 $12.00

### Book Reviews

**High Dose Rate Brachytherapy – A Textbook**
Edited by Subir Nag. New York: Futura Publishing Co. Inc., 1994. 462 pp. $69.00.

Conventional brachytherapy has always been recognised as an excellent way to deliver a high dose of radiation to the heart of a tumour but has had the disadvantage that it takes a long time and involves risk of radiation exposure. Recent developments in afterloading technology and source design now make it possible to give a high dose of radiation in a very short time without exposure hazard and with the possibility of optimising dose distribution. This multiauthor book reviews recent experience in this rapidly growing field and should be of interest to anyone who has just started or is about to start high dose rate (HDR) brachytherapy.

Those who search for guidance on dose and fractionation regimens are not going to find it, because with the exception of a few sites there is too little experience and insufficient follow-up on which to base advice.

HDR brachytherapy has an accepted role in the management of carcinoma of the bronchus and oesophagus because it allows rapid safe treatment that cannot readily be given in any other way. It is also widely used in gynaecological cancer, but for other sites there are still very few data. Many of the clinical chapters describe the individual techniques used and the results of relatively few patients treated in selected centres in the USA. They do, however, give useful details on how to perform the implant. References are plentiful and up to date with many after 1990, but there is little attempt to place HDR brachytherapy within the context of other treatments, and many valuable European data have not been included. The strongest sections are on the new dosimetry methods that have become available and their integration with imaging and with dose optimisation. There is also a very full discussion of the potentially serious issue of quality assurance.

Most of the criticisms are because the subject is so new that many of the questions arising from it have yet to be answered. It is perhaps too soon for a definitive textbook but the data presented give a good view of the current position, even though much may change over the next few years.

DV Ash